# An ex vivo culture model of kidney podocyte injury reveals mechanosensitive, synaptopodin-templating, sarcomere-like structures

**DOI:** 10.1126/sciadv.abn6027

**Published:** 2022-08-31

**Authors:** Shumeng Jiang, Farid Alisafaei, Yin-Yuan Huang, Yuan Hong, Xiangjun Peng, Chengqing Qu, Pongpratch Puapatanakul, Sanjay Jain, Jeffrey H. Miner, Guy M. Genin, Hani Y. Suleiman

**Affiliations:** ^1^ NSF Science and Technology Center for Engineering Mechanobiology, Washington University in St. Louis, St. Louis, MO, USA.; ^2^ Department of Mechanical Engineering and Materials Science, Washington University in St. Louis, St. Louis, MO, USA.; ^3^ Department of Mechanical and Industrial Engineering, New Jersey Institute of Technology, Newark, NJ, USA.; ^4^ Division of Nephrology, Department of Medicine, Washington University School of Medicine, St. Louis, MO, USA.; ^5^ Department of Cell Biology and Physiology, Washington University School of Medicine, St. Louis, MO, USA.

## Abstract

Chronic kidney diseases are widespread and incurable. The biophysical mechanisms underlying them are unclear, in part because material systems for reconstituting the microenvironment of relevant kidney cells are limited. A critical question is how kidney podocytes (glomerular epithelial cells) regenerate foot processes of the filtration apparatus following injury. Recently identified sarcomere-like structures (SLSs) with periodically spaced myosin IIA and synaptopodin appear in injured podocytes in vivo. We hypothesized that SLSs template synaptopodin in the initial stages of recovery in response to microenvironmental stimuli and tested this hypothesis by developing an ex vivo culture system that allows control of the podocyte microenvironment. Results supported our hypothesis. SLSs in podocytes that migrated from isolated kidney glomeruli presented periodic synaptopodin-positive clusters that nucleated peripheral, foot process–like extensions. SLSs were mechanoresponsive to actomyosin inhibitors and substrate stiffness. Results suggest SLSs as mechanobiological mediators of podocyte recovery and as potential targets for therapeutic intervention.

## Teaser

Patterned hydrogels reveal mechanosensitive nanotemplates in kidney podocytes for repairing disrupted cytoskeletal networks.

## INTRODUCTION

Kidney glomerular diseases result in damage to the kidney’s filtration apparatus and often cause chronic kidney disease and kidney failure with no cure (*1*). One barrier to devising successful treatments for these diseases is the lack of a full understanding of the biophysical mechanisms that underlie them (*2*). Glomerular disease may be associated with mechanobiological dysregulation of the components that comprise the three-layered filtration barrier: the endothelial cells, the glomerular basement membrane (GBM), and especially the podocytes (*3*). Podocytes are unique epithelial cells with hundreds of foot processes that interdigitate with those of adjacent podocytes; these are connected by slit diaphragms, unique intercellular junctions that are critical for filtration (*4*). However, the inability to study podocytes outside of their native microenvironment has left key gaps in our knowledge of how they maintain the intricate structures that enable filtration and of how injury and healing progress. To address this critical need, we developed a culture system that enables studying of podocyte injury outside of their native microenvironment.

Previous work on podocyte mechanobiology includes a wealth of studies in vitro using immortalized mouse and human podocyte cell lines (*5*–*7*) and, more recently, using primary mouse podocytes (*8*–*10*). However, this work has occurred with cells cultured on glass substrates. The physiological stiffness range of the podocyte microenvironment ranges from the ~0.74-kPa modulus of decellularized glomeruli to the ~2.4-kPa modulus of the GBM, and stiffness increases with pathologies such as diabetes (*2*, *11*). Kidney podocyte cell lines adopt a more physiological phenotype when cultured on slightly stiffer polyacrylamide (PAAm) hydrogels, in the 0.9- to 9.9-kPa range (*12*). Glass is a million-fold stiffer (*2*, *11*). Such a mismatch is known to affect cell structure and spreading (*12*, *13*) and a broad range of mechanobiological responses (*14*–*16*). To solve these problems, a range of biomimetic platforms has thus been proposed, including culturing immortalized podocytes on soft hydrogel–based (*17*, *18*) or polydimethylsiloxane (PDMS) (*12*) substrates, curved substrates (*19*), or micropatterned glass substrates (*20*) or in three-dimensional (3D) collagen gels (*21*). However, these systems do not produce the structures and cell shapes observed in vivo, partially because they fail to sufficiently reconstitute the microenvironment that podocytes require. Because of the fact that the mechano-responsiveness of podocytes in health and disease is thus largely unknown, we sought to develop a biomimetic platform that combines primary podocytes, physiologically relevant substrate stiffnesses, physiologic or pathophysiologic extracellular matrix (ECM) proteins, and physiologically relevant cell confinement.

The gap in knowledge that we sought to address relates to the mechanisms that podocytes use to repair connections to the GBM and to their adjacent podocyte neighbors. Studies of glomeruli in vivo have revealed that in mouse models of kidney diseases, podocytes contain the normally absent contractile protein myosin IIA in the basal aspect of the areas of foot process effacement, and the injured podocytes develop sarcomere-like structures (SLSs) (*22*). On the basis of these in vivo data, we have speculated that SLSs are associated with responses to mechanobiological cues associated with pathology and possibly associated with podocyte migration and healing (*10*, *23*). However, because SLSs are difficult to study in vivo because of their nanoscale size and because they have not been observed in any current in vitro systems, this hypothesis has not been possible to test. We therefore demonstrated the usefulness of our novel ex vivo culture system by testing this hypothesis and identifying mechanobiological factors that collectively regulate SLSs.

## RESULTS

### Micropatterned substrates with defined protein patterns representative of healthy and pathologic GBM

**Fig. 1.**
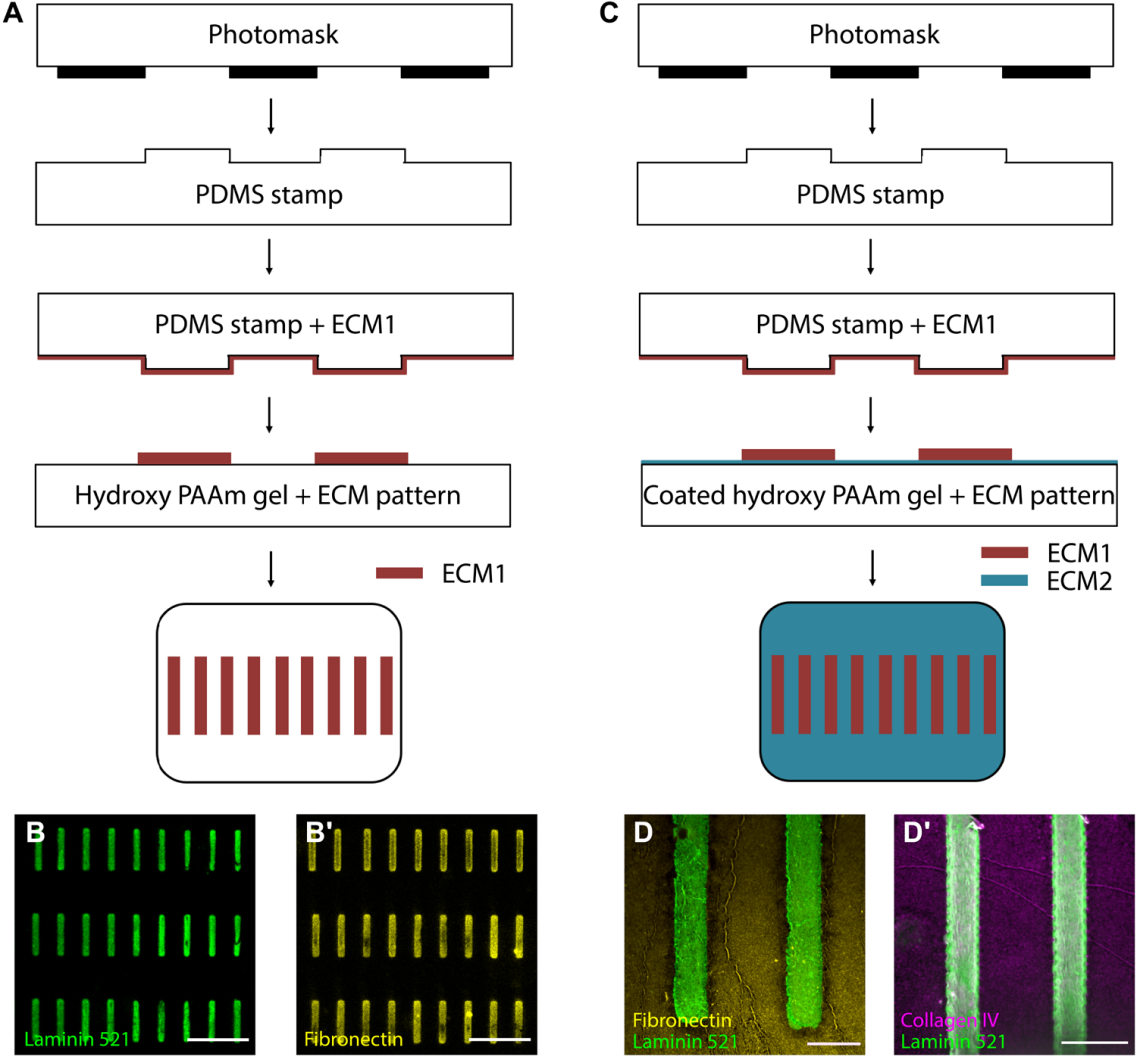
Fabrication of substrates with defined protein patterns. (**A**) Microprinting protocols for the fabrication of micropatterns with the desired ECM1 with the help of lithography. (**B**) Successfully patterned laminin 521 or fibronectin on hydroxy PAAm hydrogels. Scale bars, 50 μm. (**C**) Precoated hydroxyl PAAm hydrogel with the desired ECM2 is used instead of a plain hydrogel to enable coating both on and outside of the patterned area. (**D**) Successfully patterned hydrogels with laminin 521 on the pattern and fibronectin or collagen IV outside of the patterned area. Scale bars, 20 μm.

Using a freshly fabricated PDMS mold, we printed patterns of ECM proteins representative of the GBM in health and injury (Fig. 1A). To represent physiologic GBM, we used a PAAm hydrogel of defined stiffness to microprint human laminin α5β2γ1 trimers (Lam-521), the dominant laminin trimer in the GBM (*24*). To represent injury, Lam-521 was replaced with fibronectin, a GBM protein that is synthesized in certain glomerular diseases and podocyte injuries (*25*). Using immunofluorescence staining of the hydrogel with antibodies specific to the different ECM proteins, patterns were clearly visualized as 105 μm by 7 μm microprints (Fig. 1B). To microprint two different ECM substrates, we coated the hydrogel with the first ECM protein and let it dry before microprinting the second ECM protein atop the first (Fig. 1C). In this way, Lam-521 micropatterns were printed atop PAAm hydrogels that were coated with either collagen IV, representative of a healthy GBM, or fibronectin, representative of abnormal GBM (Fig. 1D). Note that the lines of protein condensation associated with drying of the surface proteins did not appear to affect the mechanics of the cellular microenvironment, as evident from strain fields associated with cellular contraction (fig. S1).

### Podocytes from isolated glomeruli migrate onto micropatterned Lam-521

**Fig. 2.**
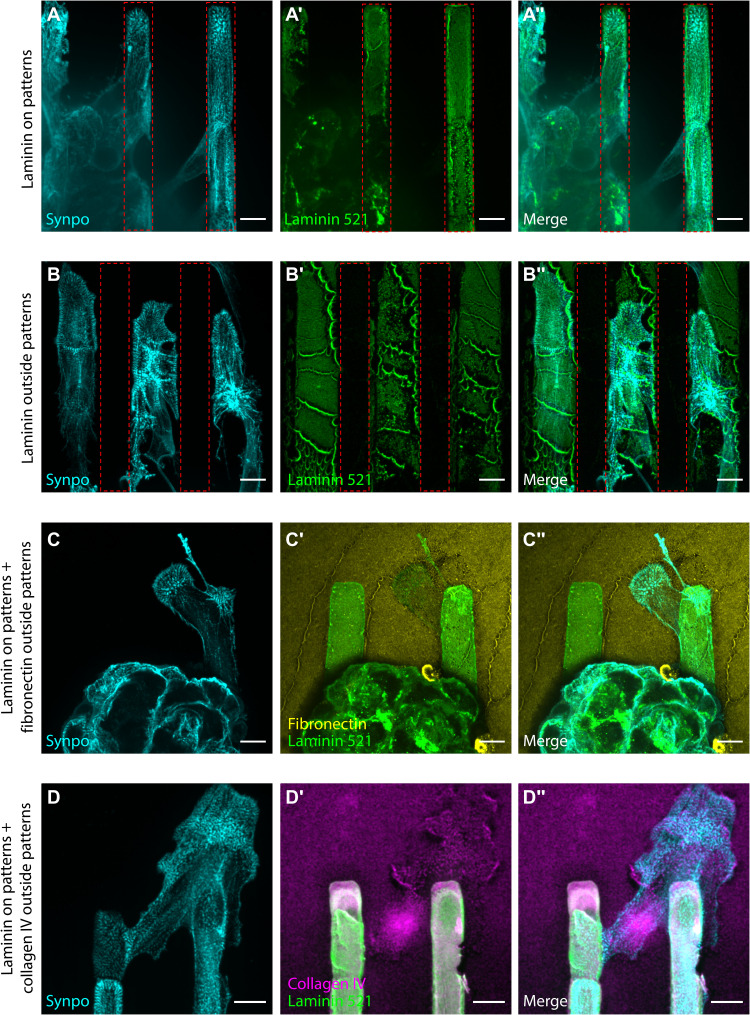
Micropatterns with combined ECM proteins reveal different stages of podocyte spreading. (**A**) Spreading primary podocytes from an isolated mouse glomerulus can be found in the patterned area where Lam-521 is presented (marked with red boxes). (**B**) A hydrogel precoated with Lam-521 and printed with a blank stamp shows Lam-521 outside of the patterned area (marked with red boxes). Spreading podocytes are found only on the Lam-521. (**C**) Podocytes spread along the micropatterned Lam-521 before sending out protrusions on the fibronectin-enriched area outside the micropatterns. (**D**) Primary podocytes populated the Lam-521 micropatterned area first and then spread onto the collagen IV–coated area. All scale bars, 10 μm.

To test which micropatterned ECM proteins best support podocytes, we isolated mouse glomeruli and cultured them on hydrogels coated with Lam-521, collagen IV, or fibronectin. Culturing the glomeruli for 2 days followed by immunostaining using antibodies against synaptopodin, a podocyte-specific cytoskeletal marker, and human laminin α5, to identify the location of the Lam-521 micropatterns (Fig. 2), revealed that the podocytes populated micropatterns coated with Lam-521 (Fig. 2A) but not those coated with fibronectin (fig. S2). Furthermore, we coated the PAAm hydrogel with Lam-521 and micropatterned protein-free patches onto the substrate using a PDMS stamp that removed Lam-521, leaving an inverse of the previous Lam-521 micropatterns interdigitated with microscale patches of exposed PAAm. Using this setup to culture glomeruli showed that podocytes migrated onto the Lam-521–coated areas but not the bare micropatterned PAAm hydrogel areas (Fig. 2B, areas outside of the red boxes).

Next, we used PAAm hydrogels coated with fibronectin and micropatterned with Lam-521. As expected, glomeruli attached only to the Lam-521 micropatterns, and podocytes only migrated onto them, whereas other cell types readily spread on both substrates (fig. S3). Podocytes could extend protrusions into regions covered with fibronectin (Fig. 2C) but only after they migrated onto the Lam-521 micropatterns. Sometimes, they migrated across regions of fibronectin to neighboring Lam-521 micropatterns (fig. S4). Similar results were observed for collagen IV–coated PAAm hydrogels with Lam-521 micropatterns, with most of the podocytes confined to the Lam-521 micropatterned regions; podocytes occasionally extended processes onto collagen IV from their Lam-521 foundations (Fig. 2D).

### Primary podocytes present with a mat of SLSs on micropatterned substrates

**Fig. 3.**
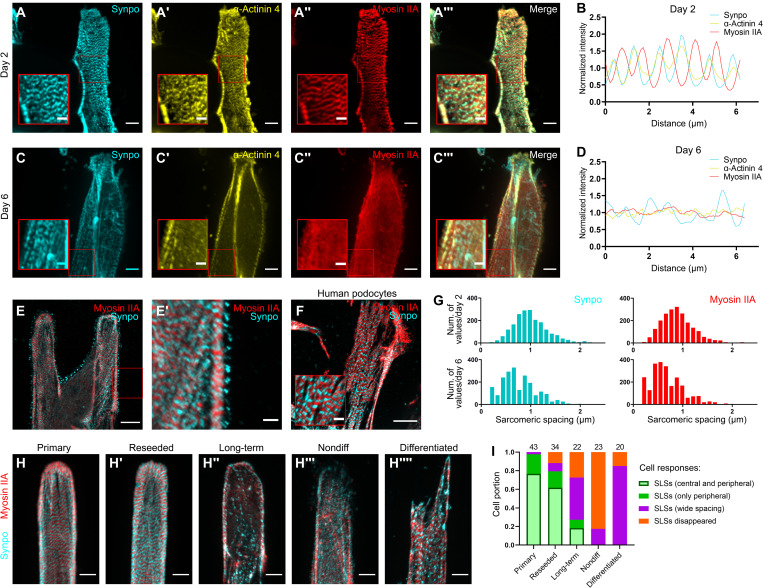
SLSs direct the early-stage spreading of primary podocytes. (**A**) SLSs could be identified after 2 days of culture, with alternating synaptopodin (which colocalized with α-actinin 4) and myosin IIA along the fibers. Scale bars, 5 μm (overview) and 2 μm (zoom-in view). (**B**) Fluorescence intensity mapping along the fibers’ direction shows myosin IIA alternating with colocalized synaptopodin and α-actinin 4. (**C**) Alternating α-actinin 4 and myosin are attenuated after 6 days of culture. Scale bars, 5 μm (overview) and 2 μm (zoom-in view). (**D**) The intensity map confirms the attenuated pattern shown in (C). (**E**) Foot process–like structures sent by the cells observed in cultured podocytes, which are positive for synaptopodin, and linked to the synaptopodin-positive band templated by the SLSs. Scale bars, 10 μm (overview) and 2 μm (zoom-in view). (**F**) SLSs could be found in human primary podocytes after 3 days of culture. Scale bars, 10 μm (overview) and 2 μm (zoom-in view). (**G**) Histogram of sarcomeric spacing represented by both synaptopodin and myosin IIA signals shows a uniform distribution of around 0.8 to 1 μm after 2 days of culture. The spacing was perturbed after 6 days of culture. *n* = 2009, 2089, 2129, and 2331 for Synpo/day 2, Synpo/day 6, Myosin IIA/day 2, and Myosin IIA/day 6. (**H** and **I**) Representative images (H to H⁗) and cell counting (I) of spreading podocytes on micropatterned hydrogels immunostained for synaptopodin and myosin IIA show the attenuation of SLSs over time in the primary podocytes [compare (H) and (H′) to (H″)]. In contrast, undifferentiated (H‴) and differentiated (H⁗) immortalized podocytes show no clear SLS patterns. Scale bar, 5 μm.

SLSs have been described in injured podocytes in vivo (*22*) but have never been observed in cultured podocytes. Immunostaining of primary podocytes migrating out of the glomeruli onto the micropatterned hydrogels showed high numbers of SLSs 2 days after seeding of glomeruli onto the Lam-521 micropatterns (Fig. 3 and fig. S5). These contained two markers of contractile activity: (i) the motor protein myosin IIA and (ii) synaptopodin, an actin-binding protein present in podocytes, arranged in a sarcomeric pattern of alternating synaptopodin and myosin IIA (Fig. 3A). α-Actinin 4, the major α-actinin isoform present in podocytes, was present in a striated pattern in registry with synaptopodin (Fig. 3A). This periodicity was confirmed by quantifying fluorescence intensity along an axis paralleling the Lam-521 micropatterns (Fig. 3B). We next asked whether these spread podocytes still have slit diaphragm proteins. An antibody against nephrin showed a lack of nephrin at the cell membrane (fig. S6), perhaps due to the fact that the cells are confined to the micropattern but require cell-cell contact to up-regulate slit diaphragm protein expression.

To investigate whether the primary podocytes that up-regulate SLSs acquire muscle-related characteristics, we immunostained the cultured podocytes for α–smooth muscle actin (α-SMA). We detected no α-SMA in podocytes, although other glomerular cell types were positive for α-SMA (fig. S7). This lack of α-SMA suggests that spreading primary podocytes presenting SLSs do not acquire muscle-related characteristics.

### SLSs are transient and can form without synaptopodin

We next asked whether SLSs represent a transient or a permanent structure in podocytes. To address this, we followed podocytes over a prolonged culture interval of 6 days. When compared to the 2-day cultures, the SLSs in 6-day cultures showed a less pronounced synaptopodin pattern and a nearly dissolved myosin IIA or α-actinin 4 pattern (Fig. 3B and fig. S5), as evident from the spatial intensity map (Fig. 3D) and from the histogram of sarcomeric spacing (Fig. 3G) or cell sorting (Fig. 3I). This suggests that the SLSs became attenuated over time.

Next, we evaluated the role of synaptopodin in the formation of SLSs. Using glomeruli isolated from *Synpo*^−/−^ mice (*10*) to culture podocytes that were then stained with α-actinin 4 and myosin IIA antibodies, we could observe SLSs in the spreading podocytes with a striated pattern similar to that of wild type. This indicates that synaptopodin is not required for formation of SLSs (fig. S8).

### SLSs are associated with newly formed peripheral foot process–like protrusions

To assess the function of the SLSs, we evaluated their role in establishing connectivity to other cells. Using two ECM proteins, Lam-521 on the micropatterns and fibronectin outside the micropatterned area, we observed that podocytes with SLSs sent out protrusions extending over areas coated with fibronectin (Fig. 2C and fig. S4). Some processes extended to connect podocytes across fibronectin patterns that were several cell widths apart (fig. S4). Moreover, the periodic synaptopodin patterning in the SLSs was associated with the spacing between the newly formed synaptopodin-positive protrusions, speculated to be immature foot process–like structures (Fig. 3E).

To confirm that SLSs are relevant to human podocytes, we applied our protocols to freshly harvested glomeruli taken from human kidney nephrectomy samples and seeded them onto the Lam-521 micropatterned hydrogels. Similar to the mouse glomeruli, human glomeruli attached to the Lam-521 micropatterns, although they took longer to attach when compared with mouse glomeruli. After 3 days of culture, sarcomeric structures could be observed in podocytes migrating from human glomeruli (Fig. 3F), confirming that SLSs are present in primary human podocytes when migrating onto the micropatterns.

### SLSs are specific to primary podocytes

Similar to the primary podocytes at day 2, reseeded primary podocytes (see Materials and Methods) maintained synaptopodin- and myosin IIA–positive SLSs (Fig. 3, H and I). This experiment suggests that formation of SLSs is a stable characteristic of primary podocytes regardless of the culture conditions. We next evaluated whether the immortalized mouse podocyte (IMP) cell line presents with SLSs. While undifferentiated IMPs showed no clear SLS patterns, differentiated IMPs [using VRAD (vitamin D3, retinoic acid and dexamethasone-supplemented DMEM/F12) differentiation media for 7 days] presented with loose synaptopodin^+^/myosin^+^ actin cables (Fig. 3, H and I). In contrast to the SLSs in primary podocytes, the distances between the periodic synaptopodin patterns in the differentiated IMPs were notably larger, and myosin no longer showed the same striated pattern (fig. S9).

### SLSs are mechanosensitive

**Fig. 4.**
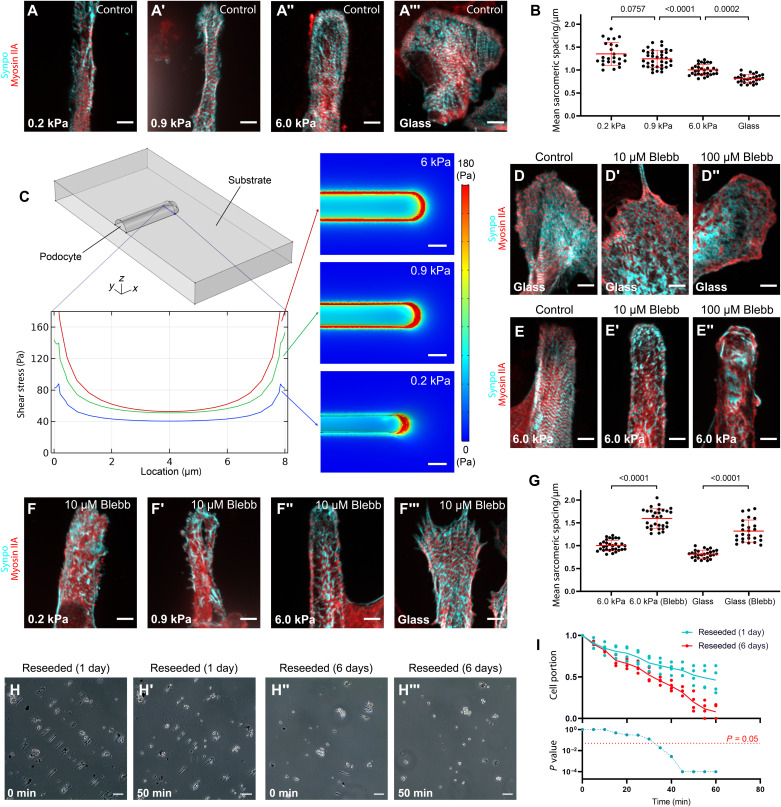
Podocyte SLSs are dependent on contractility and are sensitive to stiffness. (**A** and **B**) The uniformly distributed 1-μm-long SLSs elongated to 1.4 μm when the substrate stiffness was decreased from 6.0 to 0.2 kPa. In contrast, the extremely stiff glass substrate shortened this spacing to 0.8 μm. *n* = 25, 36, 31, and 28 for 0.2 kPa, 0.9 kPa, 6.0 kPa, and glass. Scale bars, 5 μm. (**C**) Modeling of cell contractility on different substrates shows increased contractility on stiffer hydrogels. The graph depicts predictions of the shear stress component σ*_zx_* acting in the *x* direction on the *xy* plane, along the line in the *y* direction that is pictured. Contours are of maximum principal Cauchy stress. Scale bars, 5 μm. (**D** and **E**) Myosin inhibition by blebbistatin caused a marked loss of SLSs inside podocytes cultured either on glass (D) or on patterned hydrogels (E). Scale bars, 5 μm. (**F**) Combining the effect of myosin inhibition with that of a softer substrate caused the loss of SLSs with a lower concentration of blebbistatin. Scale bars, 5 μm. (**G**) For podocytes on the stiffest substrate, myosin inhibition led to wider sarcomeric spacing. *n* = 31, 30, 28, and 26 for 6.0 kPa, 6.0 kPa (Blebb), glass, and glass (Blebb). (**H** and **I**) Detachment assay of reseeded primary podocytes with SLSs (1 day after reseeding) shows significantly less detachment compared to podocytes with attenuated SLSs (6 days after reseeding) for longer durations. Scale bars, 100 μm.

We next asked whether SLSs are sensitive to their mechanical microenvironment. PAAm hydrogels with elastic moduli in the physiologic range (0.2 and 0.9 kPa) and in pathophysiologic ranges (6.0 kPa, representative of GBM stiffening under hypertension or glycation, and cover glass, as a fully rigid substratum) were micropatterned or coated with Lam-521. After a 2-day culture of glomeruli, SLSs were evident in podocytes on the substrata of all moduli, even without the micropatterns (fig. S10), but the spacing of the sarcomeric patterning was wider on the softer hydrogels than on the stiffer hydrogels and cover glass (Fig. 4A). The periodic synaptopodin distribution from 120 cells over three replicate experiments showed substantial differences between softer hydrogels (0.2 or 0.9 kPa) and stiffer hydrogels or glass (Fig. 4B). As an additional control, we also cultured 3T3 mouse fibroblasts on hydrogels of two different stiffnesses. Similar to podocytes, 3T3 cells presented with more stress fibers on the stiff hydrogel (i.e., 6.0 kPa) than on the soft hydrogel (i.e., 0.2 kPa) (fig. S11).

### SLSs require actomyosin contractility

Given that mechanosensitivity and mechanosensing in other cell types arise from actomyosin contractility (*26*–*29*), we next asked whether SLSs can be controlled by modulating myosin II using the drug blebbistatin. In cells such as smooth muscle cells and fibroblasts, reductions in substrate stiffness reduce the resistance to actomyosin contractility and can thereby destabilize stress fibers and focal adhesions that require force for stability (*30*–*32*).

Our numerical simulations revealed that stresses were highest along the periphery of cells and at their distal ends (Fig. 4C). Stresses also increased with substrate modulus (Fig. 4C) and were attenuated by reduced cell contractility (fig. S12). Following a standard model of mechanosensitive stabilization and growth of stress fibers, these simulations thus predicted that stress fibers disassemble in the interior of cells and at the proximal ends when actomyosin contractility is attenuated either by actomyosin inhibition or by reducing substrate modulus. Simulations also predicted that cell width decreases with decreasing substrate modulus (Fig. 4C).

To test these predictions, we first applied two concentrations of blebbistatin to podocytes cultured on substrates with high stiffness (6.0-kPa PAAm hydrogels and cover glass), micropatterned or coated with Lam-521. Myosin II inhibition via blebbistatin disrupted the sarcomeric structures of SLSs in a way that depended on the dose, substrate stiffness, and position (Fig. 4, D and E), consistent with the model predictions. On both substrates, 10 μM blebbistatin caused aggregation of synaptopodin in the center of the cells and increased sarcomeric spacing in SLSs that persisted at the cell periphery (Fig. 4, D and E), as predicted by the model. Higher doses (100 μM) of blebbistatin nearly eliminated SLSs (Fig. 4, D and E). Further experiments applied a milder myosin inhibition using 2 μM blebbistatin to verify this shear lag effect. This was insufficient to disrupt the SLSs at the cell periphery but sufficient to disrupt those in the centers of the cells, and the corresponding distribution of SLSs in podocytes followed the model’s predictions (fig. S13).

A second prediction of our model was that soft substrates lead to lower cell contractility and thus enhance the effects of actomyosin inhibition. To test this, we explored whether a minimum stiffness threshold was required for the formation of SLSs in cells treated with 10 nM blebbistatin. In podocytes treated with 10 nM blebbistatin and cultured on 6.0-kPa hydrogels or glass substrates, SLSs formed but with wider sarcomeric spacing (Fig. 4, F and G). When this treatment was applied to podocytes cultured on 0.2- or 0.9-kPa hydrogels, representative of understressed GBMs under hypotensive conditions due to the nonlinearity of GBM (*33*), SLSs failed to form, with no sarcomeric structures evident (Fig. 4F). This supported the hypothesis that actomyosin-based mechanosensation governs the formation of SLSs and that a threshold of developed tension is required.

Last, our model predicted that cells would contract laterally on substrates of lower stiffness: The reduced contractile stresses associated with low substrate stiffness would be outweighed by the decreased resistance to lateral contraction. This prediction was borne out by our experiments, with cells showing narrower bodies on substrates of lower modulus (Fig. 4A).

### SLS formation can be rescued through removal of actomyosin inhibition

To test the hypothesis that mechanobiological factors alone can drive SLS formation, we asked whether SLSs susceptible to actomyosin inhibition are reversible after washing out blebbistatin. After 2 hours of inhibition, cells were left to recover for another 2 hours or 1 day and then stained for synaptopodin and myosin IIA. SLSs appeared as early as 2 hours following the removal of blebbistatin, although some condensates of synaptopodin were still evident. After 1 day of recovery, these condensates disappeared, and SLSs returned (fig. S14).

### Contractile SLSs promote podocyte adhesion to the underlying substrate

A potential role for SLSs in vivo is to enable podocytes to maintain their adhesion to the GBM even when faced with the complex and changing mechanical forces within the glomerular microenvironment. The contractile nature of SLSs is likely paramount for this role. To directly study podocyte contractility, we used reseeded primary podocytes, as more than 70% contain SLSs (Fig. 3I). Video recording of podocytes after disrupting their actin network with cytochalasin D (CytoD) showed significant cell relaxation after adding the drug. This suggests that the cells were under contractile strain before actin depolymerization. The movements of fluorescently labeled microbeads embedded in the hydrogel with either 0.9- or 6-kPa stiffnesses allowed us to calculate the strength of cell contractility. As shown by the spatial variation of the first principal Green-Lagrange strain in the adhesion plane (fig. S1), most cells relaxed and extended as contraction was attenuated by the action of the inhibitor CytoD on both stiffnesses (fig. S1). Additional strain calculation associated with the removal of cells using 10% SDS after the CytoD treatment suggests that very little strain energy is stored within the cell after CytoD treatment (fig. S1).

Last, to determine the adhesive strength of primary podocytes, we performed cell detachment assays using Cellstripper. Compared with freshly plated podocytes (i.e., 1 day after reseeding, when more than 70% have SLSs), podocytes cultured for a longer term (i.e., 6 days after reseeding, when fewer SLSs) showed higher tendencies for faster detachment (Fig. 4, H and I), suggesting an important role for SLSs in stabilizing podocyte adhesion.

## DISCUSSION

Our micropatterned culture system enabled the visualization of SLSs, which had previously been observed only in injured podocytes in vivo. Our results demonstrated the presence of SLSs in primary podocytes that had migrated from both mouse and human glomeruli and established SLSs as mechanosensitive and dynamic structures.

Our results further suggest that SLSs are associated with a healing phenotype, with SLSs linked to conditions associated with podocyte healing or antidetachment responses. Bands rich in myosin IIA were found to alternate in SLSs with bands rich in both synaptopodin and α-actinin 4. A role in healing is further suggested, because dominant mutations of α-actinin 4 are associated with diminished podocyte injury resistance, including focal segmental glomerulosclerosis (*34*). One possibility is that SLSs template periodic synaptopodin for podocyte healing and recovery, as suggested by the synaptopodin-rich processes that we observed; these are reminiscent of the foot processes that are required for normal podocyte-podocyte connectivity and podocyte function. These protrusions resembled foot processes observed during development in native podocytes, further suggesting that they are representative of a healing phenotype (*35*, *36*). Because the periodic synaptopodin patterning in the SLSs was associated with the spacing between the newly formed synaptopodin-positive protrusions, the SLSs, once laid out, might act as a template for guiding foot process formation (Fig. 3E). Although no specific pathogenic phenotype has been observed in synaptopodin knockout mice at baseline, the loss of synaptopodin exacerbates both drug-induced and genetic kidney injuries (*10*, *23*). Our ex vivo system may be useful for further clarifying synaptopodin’s roles in podocyte injury and kidney glomerular disease.

Our observation that SLS band spacing decreased with increasing substrate stiffness suggests that they are, similar to SLSs in other cells, mechanosensitive. This is consistent with observations of mechanosensitive formation of stress fibers observed in immortalized podocytes on soft gelatin substrates (*17*). The formation and organization of SLSs are also interesting: To achieve the ideal spacing in the SLSs for the templating of synaptopodin, there may be different mechanisms compared to the organization of sarcomeres in the myofibrils of muscle cells, especially considering the fact that sarcomeric spacing is usually between 1.5 and 3.5 μm in myofibrils (*37*), whereas we determined the sarcomeric spacing in SLSs to be between 0.8 and 1 μm in spreading podocytes during culturing on hydrogels with physiologic stiffness. Note that this is comparable to striated α-actinin patterns along the stress fibers of other spreading epithelial cells (*38*). The spacing between the periodic striations of sarcomeric-like stress fibers varies depending on the cell types, with ~0.5 μm in osteosarcoma cells (*39*) and ~1.6 μm in fibroblasts (*40*). The mechanisms that determine the different spacing and their relationship to the contractile forces are unknown.

In addition to templating, SLSs may serve as a buffer against mechanical perturbations that could cause podocyte detachment from the GBM and their loss into the urine. Perturbations include changes to extrinsic shear stresses associated with filtration that arise from pressure differences between the capillaries and Bowman’s space (such as in the hypertension condition), as well as changes to intrinsic stresses as may occur in response to pathological stiffening of the GBM (*36*). The sarcomeric patterns of SLSs are reminiscent of sarcomeric contractile units in muscle (*41*) and contractile nonmuscle cells (*42*), including contractile actin cables that comprise the ventral stress fibers and the transverse arcs (*43*). Similar to stress fibers in fibroblasts (*44*), SLSs in primary podocytes aligned in the direction of maximum stress, at a density that correlated with the amplitude of stress, and may serve to stabilize motor clutch bonds to the ECM (*28*). SLS alignment with the direction of cell spreading might power cell spreading and colonization of the GBM surrounding the glomerular capillaries.

The micropatterns used in this study were rectangles of approximately 20 μm in length, with length:width ratios of 14:1 to 8:1. This size range allowed the spreading of podocytes on the 2D hydrogel surfaces, while part of the podocytes remained in contact with the glomerulus. Thus, podocytes remained connected to their native 3D structure while extending into confined patterns that mimicked the constraints of the GBM. One limitation of our system is that the micropatterns limit the ability of podocytes to form foot processes and slit diaphragms, and future work should address this inadequacy. Extensions of this technology via nanopatterning may better replicate the cell microenvironment and perhaps spur the formation of foot processes. The GBM has a curvature that, although large in radius compared to the size of a podocyte foot process, may nevertheless be a factor in podocyte mechanobiology. Incorporating curvature with microprinting via surface engraving technologies may enable exploration of these factors. Densely cultured rat primary podocytes were shown to form interdigitating cell processes upon addition of heparin and all-trans retinoic acid to the media (*45*). Such an approach in combination with our culture system could allow us to study the relationship between the interdigitating foot process–like protrusions and SLSs. Last, fluid shear stresses are an additional factor that is likely important in podocyte homeostasis, and the addition of microfluidics to the system may constitute an important step forward. However, even with these limitations, our culture system has served to identify and validate essential biophysical mechanisms underlying SLSs, including mechanobiological factors that serve as potential therapeutic targets. SLSs may be a transient feature of podocyte healing or an advantageous response to injury and thus a target for intervention in kidney injury and disease.

## MATERIALS AND METHODS

### Activation of cover glasses

To enable firm attachment of PAAm hydrogels to cover glasses, the cover glasses were washed twice in NaOH (0.1 M) for 5 min, rinsed with ddH_2_O, and dried before applying a thin layer of 3-(trimethoxysilyl)propyl acrylate for 1 hour at room temperature. They were then washed again in ddH_2_O and dried under nitrogen flow (*46*).

### Preparation of hydroxy-PAAm hydrogels with defined elastic modulus

Preparation of hydroxy-PAAm hydrogels with defined elastic modulus was achieved by cross-linking precursors on activated cover glasses via modification of established protocols (*47*). Hydroxy-PAAm hydrogels were prepared using acrylamide [3.2 to 6.4% (w/w) in Hepes (pH 7.4)], bis-acrylamide [0.03 to 0.16% (w/w) in Hepes (pH 7.4)], and *N*-hydroxyethyl acrylamide (HEA) [1.3% (w/w) in Hepes (pH 7.4)] together with ammonium persulfate (APS) and *N*-tetramethylenediamine (TEMED). After incubating the precross-linking solution (i.e., the acrylamide, the bis-acrylamide, and the HEA) for 30 min under vacuum to remove oxygen and thus prevent oxidation, the cross-linking agents were added and incubated for another 30 min at room temperature. For 5 ml of precross-linking solution, 2.5 μl of TEMED and 25 μl of 10% APS were added. To shape the hydrogel as a thin layer and protect it from oxidation, a clean, nonactivated cover glass was placed on top. After the hydrogel solidified, the sample was washed three times with ddH_2_O and left at 4°C. To control the stiffness of the hydrogel, both acrylamide and bis-acrylamide final concentrations were altered, with acrylamide varied from 3.2 to 6.4% (w/w), and bis-acrylamide varied from 0.03 to 0.16% (w/w). The moduli of hydrogels were measured using atomic force microscopy (*48*) and were controlled to range in stiffness from 0.9 to 6.0 kPa, a range that encompasses that reported for GBM (*11*).

### Micropattern stamps for microprinting

A silicon master with the desired micropattern design was prepared using standard lithographic techniques (*49*). A PDMS stamp was prepared by mixing PDMS and a cross-linking agent at a 10:1 ratio. PDMS was degassed under vacuum to remove air bubbles. The molding mixture was then added to a container in the presence of the silicon master template, set inside an oven (VWR) at 60°C for 2 hours to initiate the cross-linking.

Next, the PDMS stamp was cleaned by sonication (Branson) for 20 min and washed in 50% ethanol solution before drying under nitrogen flow followed by plasma cleaning for 2 min (Harrick Plasma). Last, the PDMS stamp micropattern areas were soaked in different ECM solutions [laminin 521 (50 μg/ml; Biolamina, LN521-05), fibronectin (Corning, 356008), or collagen IV (Corning, 354233) in phosphate-buffered saline (PBS)] and left to set for 1 hour at room temperature.

After air-drying the ECM proteins atop the PDMS stamp, excess solution was removed, and the stamp was dried further using nitrogen flow. The freshly prepared hydroxyl-PAAm hydrogel was also dried out by treatment with nitrogen flow. To microprint one ECM, (i) the PDMS stamp was turned so that the patterned surface faced the hydroxyl-PAAm hydrogel surface, (ii) the stamp was gently placed onto the center of the hydroxyl-PAAm hydrogel and left there for 1 hour at room temperature, and lastly, (iii) the PDMS stamp was removed carefully, and the hydrogel was washed three times with PBS to remove unbound proteins. The linkage between the ECM protein and the hydrogel was enabled through HEA integrated into the hydrogel (*46*).

For micropatterns with combined ECM proteins, the protocol was the same except that the ECM solutions were added on top of the hydroxyl-PAAm hydrogel and left for 1 hour at room temperature before drying and the subsequent application of the above microprinting protocol. For the removal of laminin in the patterned area, a hydrophobic blank PDMS was microprinted onto the hydrogel and left for 1 hour at room temperature before removal.

### Isolation of mouse glomeruli

Animal experiments were approved by the Washington University in St. Louis Institutional Animal Care and Use Committee under protocol number 21-0089. Mouse glomeruli were isolated using an established differential adhesion method (*50*) with modifications. Briefly, kidneys were collected, minced, and digested in collagenase A (Roche, 10103586001) solution in Hanks’ balanced salt solution (HBSS) (1 mg/ml; Gibco, 24020-117) at 37°C for 15 min. The collagenase A digestion was stopped by adding an equal volume of Dulbecco’s modified Eagle’s medium (DMEM) with 10% fetal bovine serum (FBS). Next, the suspension containing the dissociated kidney fragments was passed through three differently sized cell strainers (100, 70, and then 40 μm, MIDSCI), and the glomeruli-enriched tissue fragments were collected on top of the 40-μm cell strainer. These were then placed onto 10-cm tissue culture dishes (TPP) for 1 to 2 min to allow the tubular segments to adhere before collecting the glomeruli left in suspension. For higher glomerular purity, the adhesion step was repeated twice. Last, the suspension was spun down under 290*g* for 5 min, and the glomeruli were resuspended in primary podocyte culture medium and directly used for the downstream applications.

The mouse podocyte culture medium was prepared as reported earlier (*51*). Briefly, for 648 ml of podocyte culture medium, we mixed the following: (i) 3T3L1 conditional media (300 ml), which was the supernatant arising from the culture of the 3T3L1 cell line for 3 days at 37°C in DMEM culture medium (Gibco, 11965-084) containing 10% FBS (Sigma-Aldrich, F7524) and 1% penicillin-streptomycin (Sigma-Aldrich, P4333); (ii) low-glucose DMEM (204 ml); (iii) Ham’s F-12; (iv) l-glutamine (102 ml; Lonza); (v) FBS (30 ml); (vi) penicillin-streptomycin (6 ml) (Sigma-Aldrich, P4333); and (vii) insulin-transferrin-selenium liquid media supplement (6 ml) (Invitrogen, 41400045).

### Isolation and reseeding of primary mouse podocytes

To reseed primary podocytes without glomeruli, mouse glomeruli were isolated with the assistance of magnetic microbeads (*52*). Briefly, 150 μl of Dynabeads M-450 Epoxy (Invitrogen, 01130646) was diluted into 10 ml of HBSS and used to perfuse the mice. After the perfusion, the kidneys were collected, minced, and digested in collagenase A solution in HBSS at 37°C for 15 min. Collagenase A activity was stopped by adding an equal volume of DMEM with 10% FBS. Next, the kidney suspension containing various nephron fragments including the glomeruli was passed through a 100-μm cell strainer twice and centrifuged at 200*g* for 5 min. The pellet was collected and resuspended in 3 ml of HBSS. Last, the glomeruli were collected using a magnet concentrator, washed three times before being suspended in mouse podocyte culture medium, and cultured in 10-mm culture dishes. After 3 days of culture, the glomeruli and spreading podocytes were lifted off the dish using Cellstripper (Corning, 25-056-CI) and passed through a 40-μm cell strainer to filter out the glomeruli. Last, the pure primary podocytes were reseeded for downstream experiments.

### Isolation of human glomeruli

Samples were collected by the Kidney Translational Research Center under a protocol approved by the Washington University Institutional Review Board (IRB 766 #201102312). All patients consented to the research. Kidney nephrectomy tissue was minced, passed through a 250-μm metal cell strainer (MIDSCI) using 1× HBSS solution (Gibco, 24020-117), and collected on a 70-μm cell strainer. The glomeruli-rich suspension was spun down at 2000*g* (*g* = 9.81 m/s^2^) for 5 min and rinsed by passing it through the 70-μm strainer using HBSS. Last, the glomeruli on top of the 70-μm cell strainer were rinsed and resuspended in human primary podocyte culture medium, consisting of DMEM/F12 medium (Thermo Fisher Scientific, 11320-033) with 1% (v/v) insulin-transferrin-selenium (Invitrogen, 41400045), 20% (v/v) FBS (Sigma-Aldrich, F7524), and 1% (v/v) penicillin-streptomycin (Sigma-Aldrich, P4333). These were directly used for the downstream applications (*53*).

### Glomerular culture and podocyte spreading on micropatterned hydrogels

Before culturing the glomeruli, micropatterned hydrogels were moved inside a six-well culture plate and washed thoroughly with HBSS. After the isolation, glomeruli or primary podocytes suspended in podocyte culture medium were placed on the micropatterned hydrogel. For a single hydrogel, ~100 μl of the glomerular suspension was added and cultured at 37°C, 5% CO_2_, and 95% humidity. Additional podocyte culture medium (2 ml) could be added to the culture after 10 hours of attachment for primary glomeruli and 2 hours for primary podocytes.

### Culture and differentiation of immortalized cells

IMPs were cultured on the basis of established methods (*54*, *55*). Briefly, cells proliferated under permissible conditions using RPMI medium (Gibco, 11-875-085) with 10% of FBS in the presence of recombinant mouse interferon-γ (IFN-γ) (10 U/ml; BioLegend, 575304) at 33°C. For differentiation, the medium was replaced with VRAD medium [DMEM/F-12 medium (Gibco, 11-330-032) complemented with vitamin D(3), retinoic acid, and dexamethasone] without IFN-γ, and podocytes were incubated at 37°C for 7 days (*56*). Cells under these nonpermissible conditions stop proliferating, increase their size, and grow processes during the differentiation process. Last, cells were trypsinized and seeded on micropatterned hydrogels. Immortalized 3T3 fibroblasts were cultured using DMEM supplemented with 10% FBS and 1% of penicillin-streptomycin.

### Immunofluorescence staining

After spreading, cultured podocytes on the hydrogels or glass were fixed in 4% paraformaldehyde (Electron Microscopy Sciences, 15712) for 10 min at room temperature followed by washing with 1× PBS, three times for 6 min each, and then permeabilized using 0.05% Triton X-100 for 10 min at room temperature. Next, samples were blocked using 2% bovine serum albumin (Sigma-Aldrich, A7906) for 30 min at room temperature before applying the primary antibodies overnight at 4°C. Next, samples were washed with 1× PBS, three times for 6 min each before incubating them with the secondary antibodies in PBS for 1 hour at room temperature. Last, the samples were washed in 1× PBS, three times for 6 min and prepared for mounting using Invitrogen Slow Fade mounting medium (Invitrogen, S36917). The cover glass holding the sample (hydrogel and cells) was bonded to a second cover glass using nail polish.

The primary antibodies used were myosin IIA (BioLegend, 909801, rabbit anti-mouse), myosin IIA (Abnova, clone 3C7, mouse anti-human, NH2-terminus), synaptopodin (Synaptic Systems, 163004, guinea pig anti-mouse), laminin α5 [clone 4C7, mouse anti-human, (*57*)], fibronectin (Sigma-Aldrich, F3648, rabbit anti-human), collagen IV (SouthernBiotech, 1340-01, goat anti-human), nephrin (R&D Systems, AF3159, goat anti-mouse), and α-SMA (Invitrogen, 14-9760-82). Alexa Fluor 555 phalloidin (Thermo Fisher Scientific, A34055) was used for actin staining. For mouse myosin antibodies (clone 3C7), samples were antigen retrieved in TE (Tris-EDTA) buffer (pH 9.0) at 65°C for 4 hours before the permeabilization step.

### Confocal microscopy

Imaging was performed on a Zeiss LSM 880 confocal microscope [equipped with a unique scan head incorporating a high-resolution galvo scanner along with two photomultiplier tubes (PMTs) and a 32-element spectral detector as well as a transmitted light PMT for differential interference contrast imaging] or a Nikon spinning disk confocal microscope (equipped with a Yokagawa CSU-X1 variable speed Nipkow spinning disk scan head, Andor Zyla sCMOS cameras, and a light-emitting diode–based DMD (Deformable Mirror Device) system for ultrafast photostimulation). Images were taken using a 10× objective for micropatterns with a single protein and using a 60× or 100× oil-immersive objective for other images.

### Myosin II inhibition with blebbistatin

Blebbistatin (EMD Millipore, 203389-5) was used to inhibit the function of myosin II in the podocytes. At the second day of primary podocyte culture, blebbistatin (50 mM stock solution) was added directly into the podocyte culture medium for a final concentration ranging from 2 to 100 μM. After 2 hours of treatment, the samples were washed with PBS and immediately fixed and stained as mentioned above.

### Strain mapping with microbead-embedded hydrogels

Fluorescent microbeads with a diameter of 0.2 μm (Invitrogen, F8801) were added to the hydrogel’s pre-cross-linking solution with a 1:60 dilution ratio before the cross-linking step. After solidification, hydrogels were used for the strain mapping as reported earlier (*58*). Briefly, primary mouse podocytes were isolated as described above, seeded on the hydrogels, and allowed to spread for 24 hours. Next, the cells were transferred to a live-imaging chamber before adding CytoD to the culture medium (CytoD, Sigma-Aldrich, 22144-77-0; final concentration, 20 μM). In the final step, the culture medium was changed to 10% SDS to remove the attached cells completely. Cell relaxation and microbead movements were recorded immediately after adding CytoD or 10% SDS, and the strain field was calculated using a strain mapping algorithm that was developed in our previous study (*59*).

### Numerical simulations

A finite element model of a cell process contracting isotropically atop an elastic substrate was studied. The cell and the substrate were approximated as isotropic continua. The cell had an elastic modulus of 1.0 kPa and a Poisson ratio of 0.3. The substrate had an elastic modulus that was varied from 0.2 to 6.0 kPa and a Poisson ratio of 0.3. The cell process was given the dimensions shown in Fig. 4C in which the cell shape was assumed to be half of a cylinder capped with quarter of a sphere, both with a radius of 4 μm. The underlying substrate was 100 μm in its width and length while 10 μm in thickness. The substrate base was fixed, and the sides were traction free. Convergence was achieved when the substrate was discretized with 67,354 quadratic elements and the cell with 50,573 quadratic elements. The cell’s reference configuration contracted by 10% in the *x* direction, 8% in the *y* direction, and 2% in the *z* direction, considering that the main contractility should follow the *x* direction (Fig. 3A). This contraction was resisted by elastic stresses stored in the cell and the substrate. In the simulation, different contraction parameters were used. Contraction in all the directions was decreased to 50 or 20% of their original value for medium- and low-contraction situations, respectively. Simulations were performed using Comsol (Comsol Inc., Burlington, MA).

### Quantitative microscopy of sarcomeres

Sarcomeric structures were measured using ImageJ (v1.53e with Bio-Formats plugin). Briefly, fluorescence intensity maps were acquired by plotting the intensity along a line drawn perpendicular to the sarcomere structure. For histogram, the spacing of each sarcomere is calculated by recording the peak location in the intensity map, and *n* for each group represents the total sarcomere number calculated in that group. For averaged sarcomeric spacing, the mean values were calculated for each intensity map (i.e., each stress fiber that is no less than 6 μm long), while *n* for each group represents the number of stress fibers analyzed in that group.

### Cell counting and scoring

For counting and scoring cell phenotypes, imaged podocytes were divided into several categories, including (i) cells with SLSs centrally and in the periphery, (ii) cells with SLSs only in the periphery, (iii) cells with loose striated patterns of synaptopodin and myosin IIA instead of a mat of SLSs (i.e., widely spaced SLSs), and, lastly, (iv) cells without SLSs. For measuring cell length, the longest axis of the cell was determined (mostly along the micropattern direction), and the length was determined through ImageJ (v1.53e with Bio-Formats plugin).

### Statistical analysis

Significance analysis for sarcomeric spacing was performed using one-way analysis of variance (ANOVA) followed by Tukey’s multiple comparisons test with the GraphPad Prism version 9.0 (GraphPad Software, San Diego, CA). Significance analysis for detachment assay was performed using two-way ANOVA followed by Šídák’s multiple comparisons test with GraphPad Prism version 9.0. For all data, differences were represented by a *P* value that was available upon each of the analyzed data. Data were expressed with each data point when available and together with means or means ± SD.

## Notes

View/request a protocol for this paper from *Bio-protocol*.
