# Supplementary material for: An ex vivo culture model of kidney podocyte injury reveals mechanosensitive, synaptopodin-templating, sarcomere-like structures
Source: Sci Adv. 2022 Aug 31;8(35):eabn6027. doi: 10.1126/sciadv.abn6027 (PMC9432837; doi:10.1126/sciadv.abn6027)
Supplement: Supplementary file 1 — Figs. S1 to S14 [file sciadv.abn6027_sm.pdf]

Science Advances
AAAS

Supplementary Materials for

An ex vivo culture model of kidney podocyte injury reveals mechanosensitive, synaptopodin-templating, sarcomere-like structures

Shumeng Jiang *et al.*

Corresponding author: Hani Y. Suleiman, hsuleiman@wustl.edu; Guy M. Genin, genin@wustl.edu; Jeffrey H. Miner, minerj@wustl.edu

Sci. Adv. **8**, eabn6027 (2022)
DOI: 10.1126/sciadv.abn6027

This PDF file includes:

Figs. S1 to S14

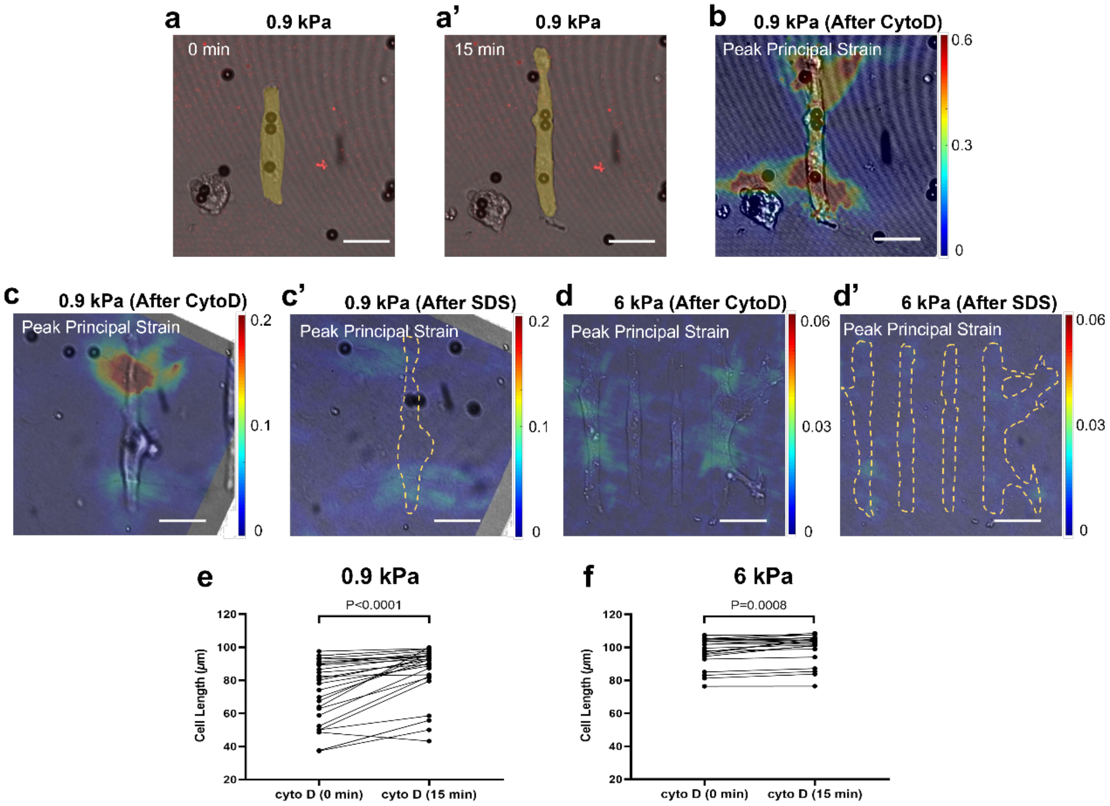

Fig. S1.

Evidence that podocytes were contractile. (a) Upon disruption of the actin cytoskeleton and inhibition of further actin polymerization using cytochalasin D (CytoD), most cells elongated along their axes, indicated that they were in a state of contraction. Shown here is a cell on a 0.9 kPa substrate just before (a) and 15 min after (a’) application of CytoD. (b) Strain mapping based on fluorescent microbeads embedded within the 0.9 kPa hydrogel revealed that podocytes were contractile. The contour plot shows the peak eigenvalue of the Green-Lagrange strain tensor field associated with movement of beads as cells released tension over 15 minutes following treatment with CytoD on the 0.9 kPa hydrogels shown. Strains shown take the post-treatment state (a’) as the reference configuration. (c) To verify that the contraction was predominantly associated with contractility in the actin cytoskeleton, an additional strain mapping was performed following elimination of the cells using the detergent sodium dodecyl sulfate (SDS). The strains associated with this subsequent SDS treatment were very small compared to those associated with CytoD. (d) Strains associated with contraction transmitted through actin cytoskeleton, calculated as in (b) and (c), were smaller on stiffer (6 kPa) substrates. Again, strains associated with CytoD treatment were much larger than those associated with subsequent SDS treatment. (e) Paired t-tests revealed that the increases in cell length associated with 15 min of CytoD treatment were statistically significant. (f) Less cell relaxation is recorded on a hydrogel with a stiffness of 6 kPa (f). Scale bar, 20 μm.

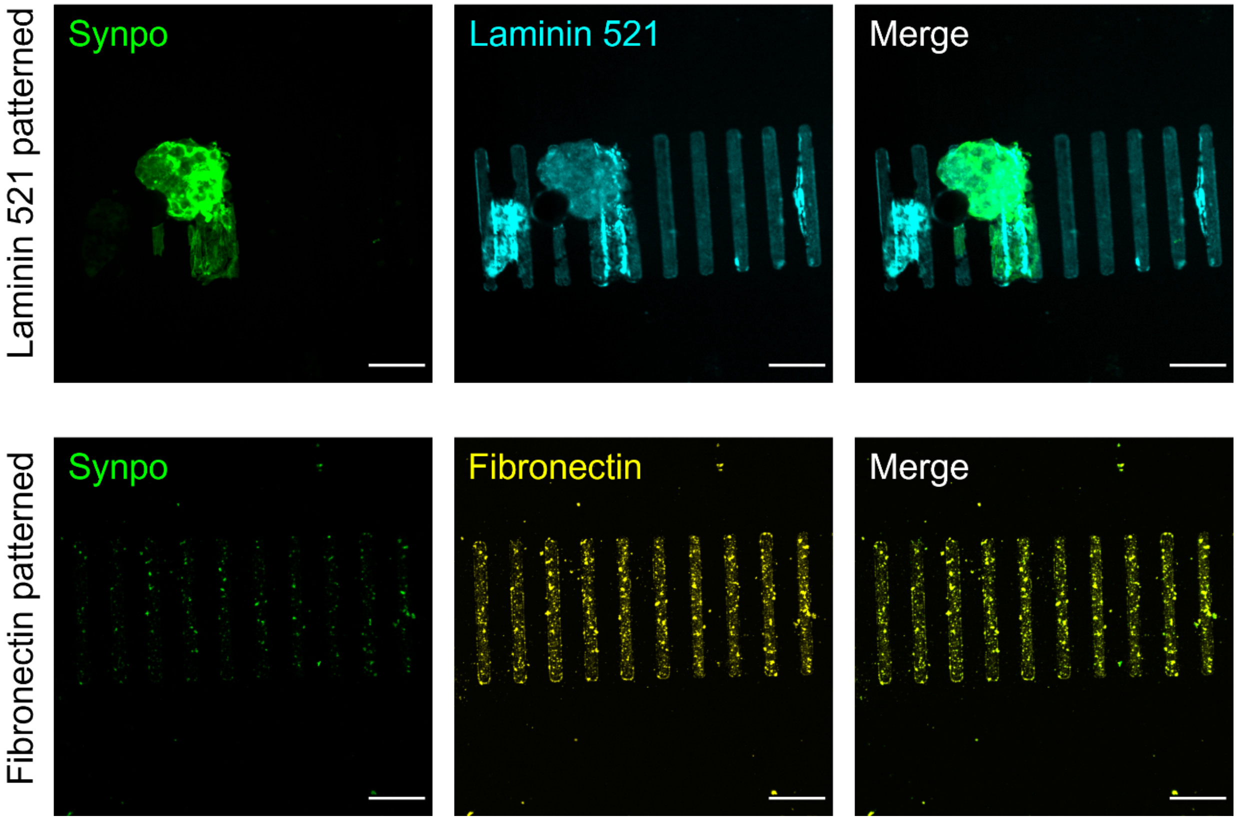

Fig. S2.

Unlike Laminin 521 micropatterns, Fibronectin micropatterns are not efficient at enabling glomerulus/podocyte attachment on the hydrogels. Scale bars, 50 µm.

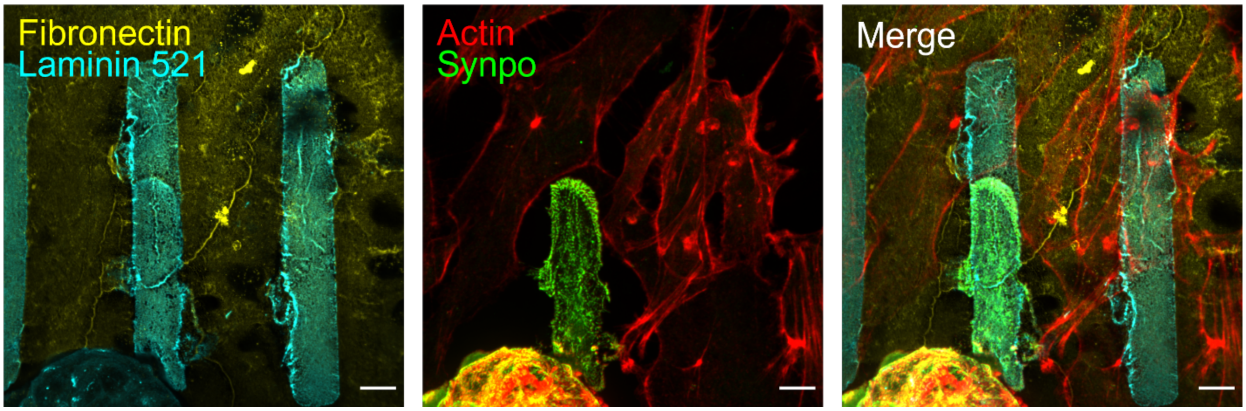

Fig. S3.

The preferential adhesion/migration on laminin-521 is specific for podocytes, as there are many cells without synaptopodin attached to the fibronectin between the laminin-521 micropatterns. Scale bars, 10 µm.

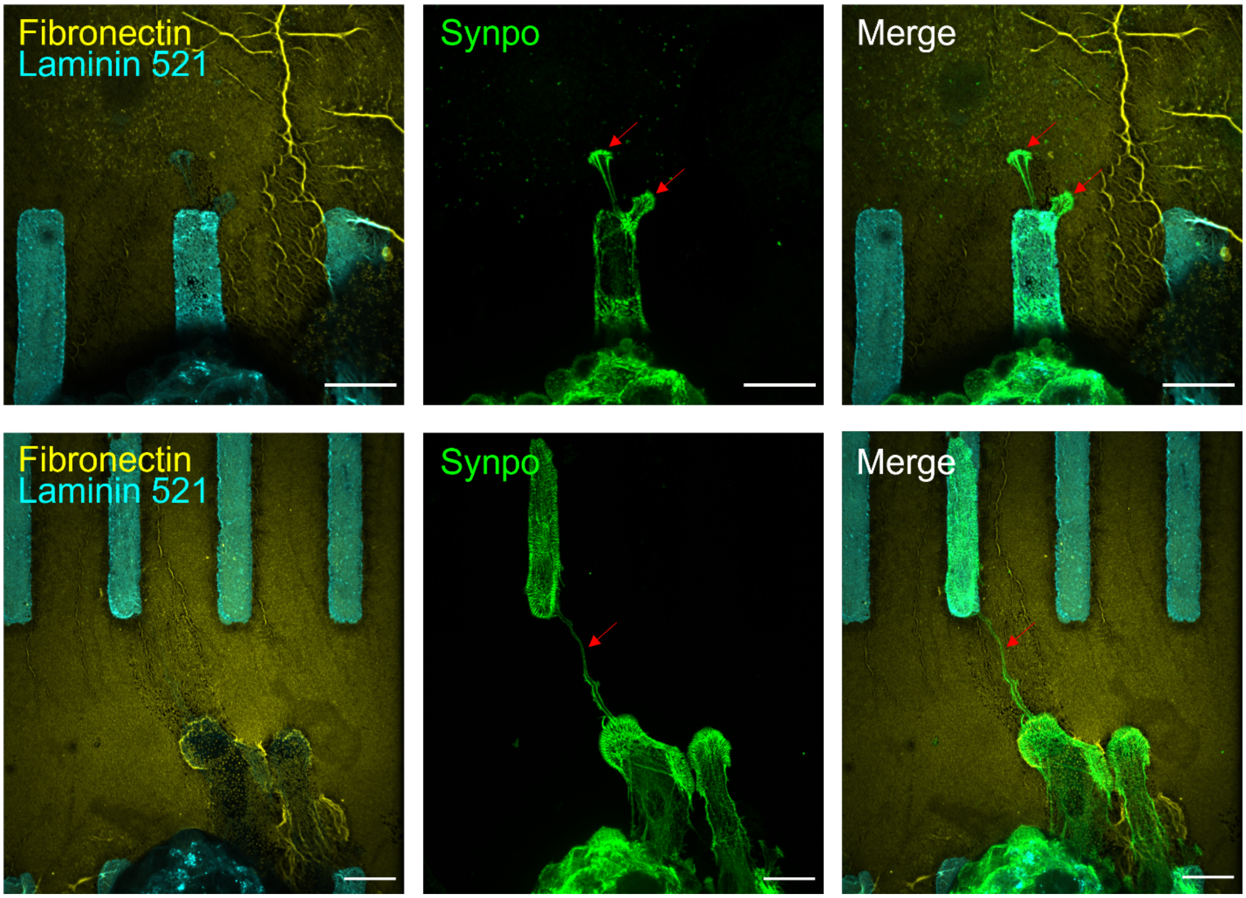

Fig. S4.

When cultured on Laminin 521-micropatterned hydrogels with Fibronectin outside the micropatterns, the podocytes send out synaptopodin-positive protrusions (Marked by red arrows); these seem to enable migration to a nearby micropattern. Scale bars, 20 µm.

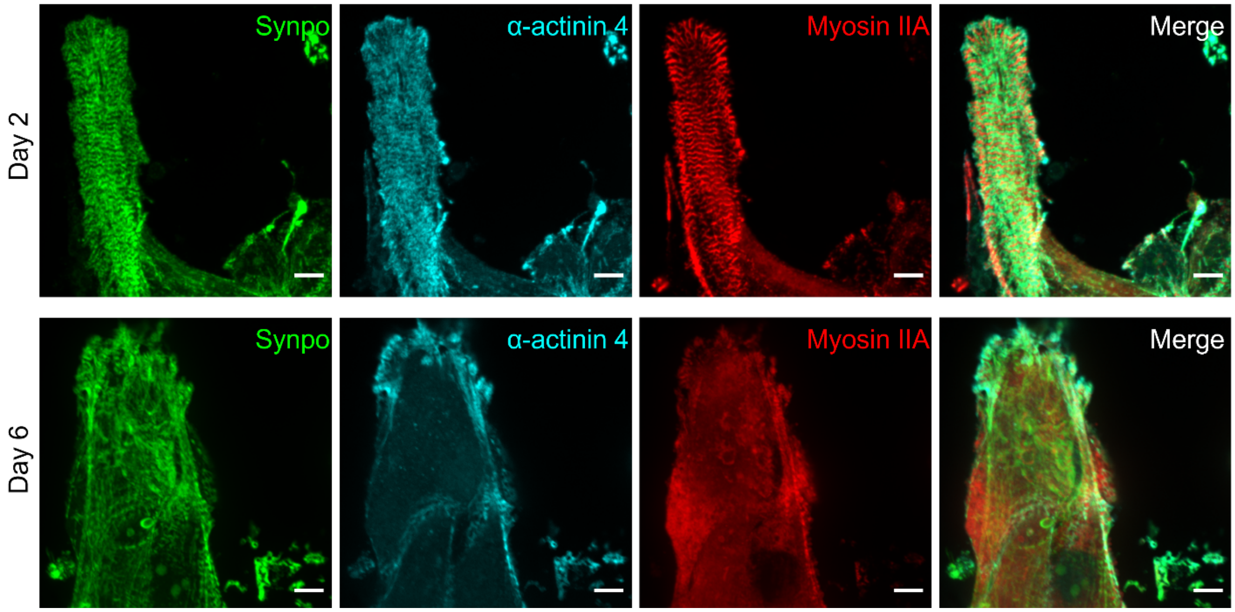

Fig. S5.
Additional images show podocytes with SLSs on day 2, but most of them are lost by day 6. Scale bars, 5 μm.

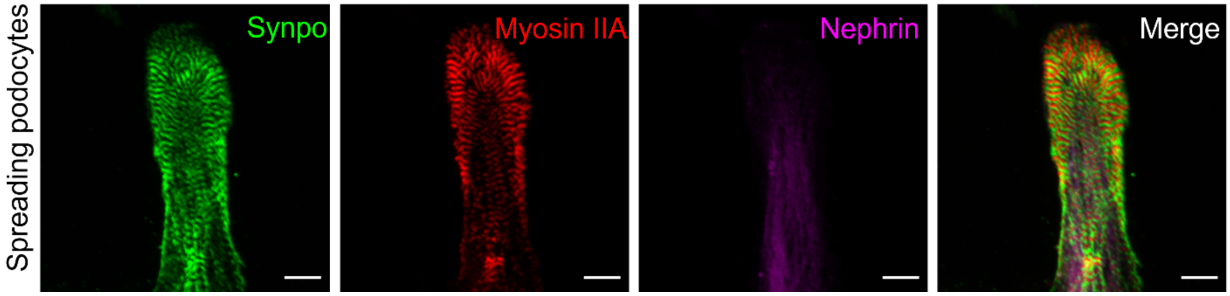

Fig. S6.

Immunostaining for nephrin in spreading primary podocytes shows no significant accumulation of nephrin. Scale bars, 5 μm.

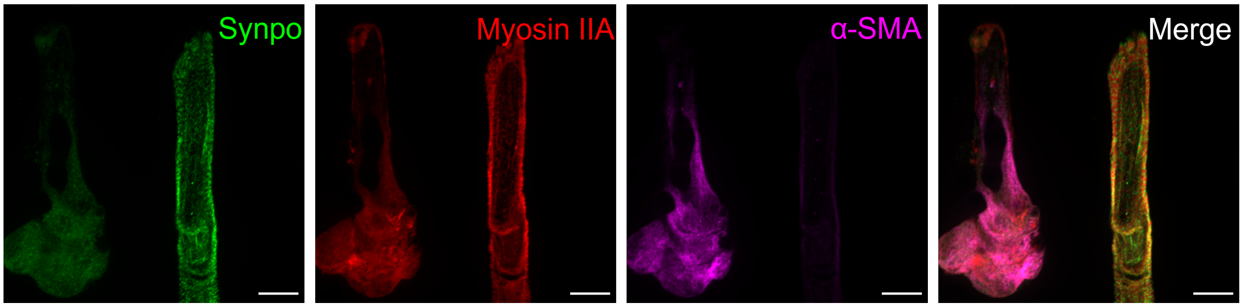

Fig. S7.
Immunostaining for α-SMA in spreading primary podocytes shows no signal in synaptopodin-positive cells with SLSs. However, α-SMA is present in non-podocyte cells identified by negative synaptopodin immunostaining. Scale bars, 10 µm.

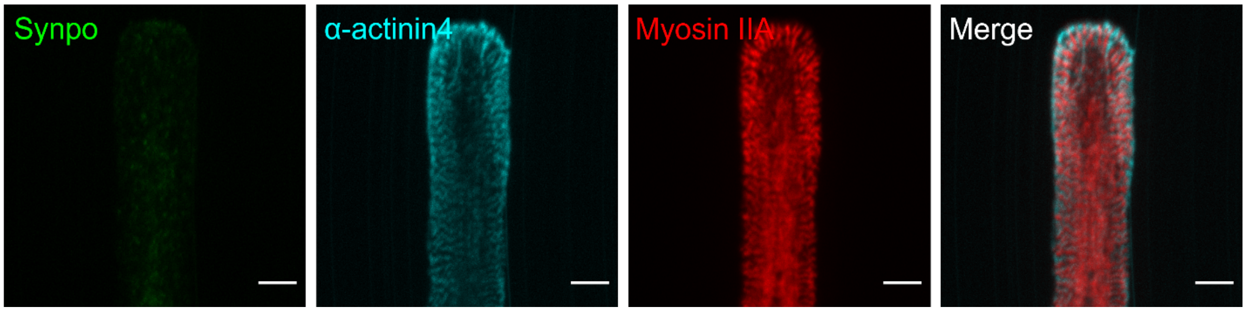

Fig. S8.
Synaptopodin, α-actinin4 and myosin IIA staining of spreading podocytes isolated from *Synpo*$^{-/-}$ mice. While no synaptopodin signal was present, podocytes still presented with striated SLS patterns identified as alternating α-actinin4 and myosin IIA staining. Scale bars, 5 µm.

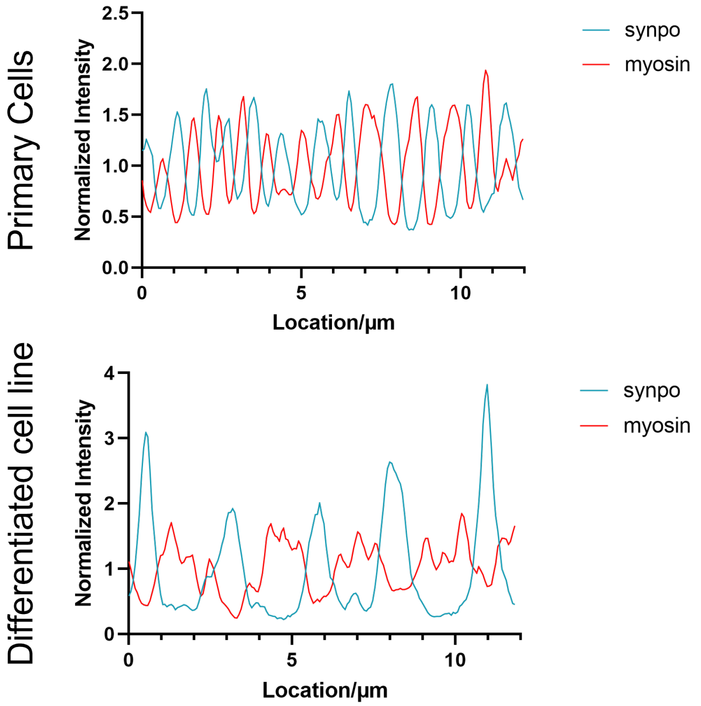

Fig. S9.

In differentiated immortalized podocytes, the SLSs showed dramatically larger spacings between synaptopodin positive bands compared to primary podocytes, whereas myosin showed multiple bands in between synaptopodin bands.

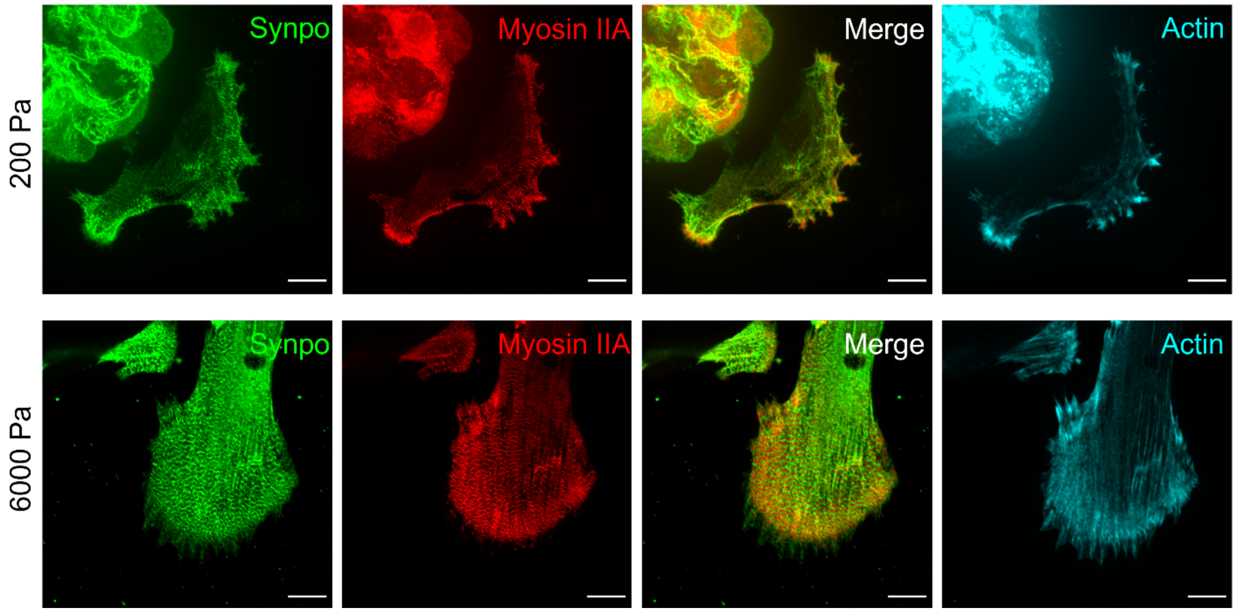

Fig. S10.
Primary podocytes spread on non-patterned soft (200 Pa) and stiff (6000 Pa) hydrogels coated with laminin-521 both show SLS patterns. Scale bars, 10 μm.

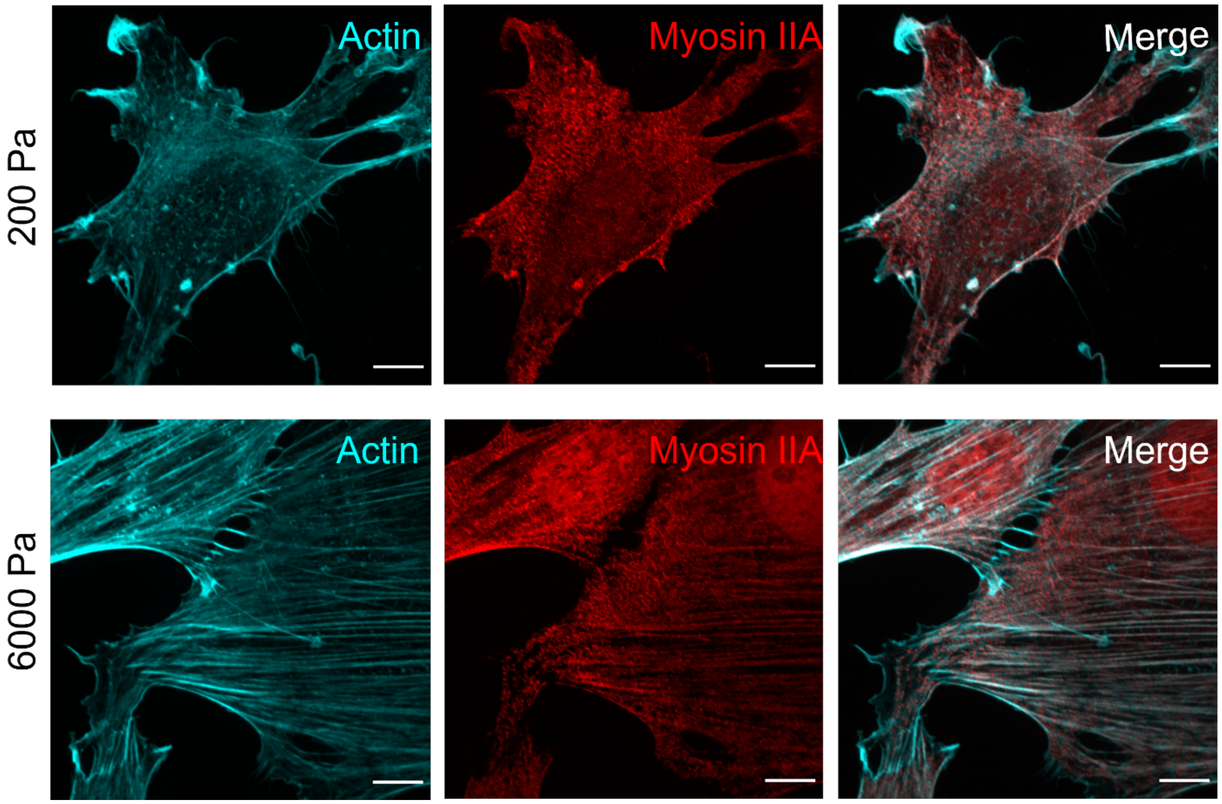

Fig. S11.
3T3 cells cultured on hydrogels of different stiffnesses showed significantly more stress fibers on the 6000 Pa substrate vs. the 200 Pa substrate. Scale bars, 10 µm.

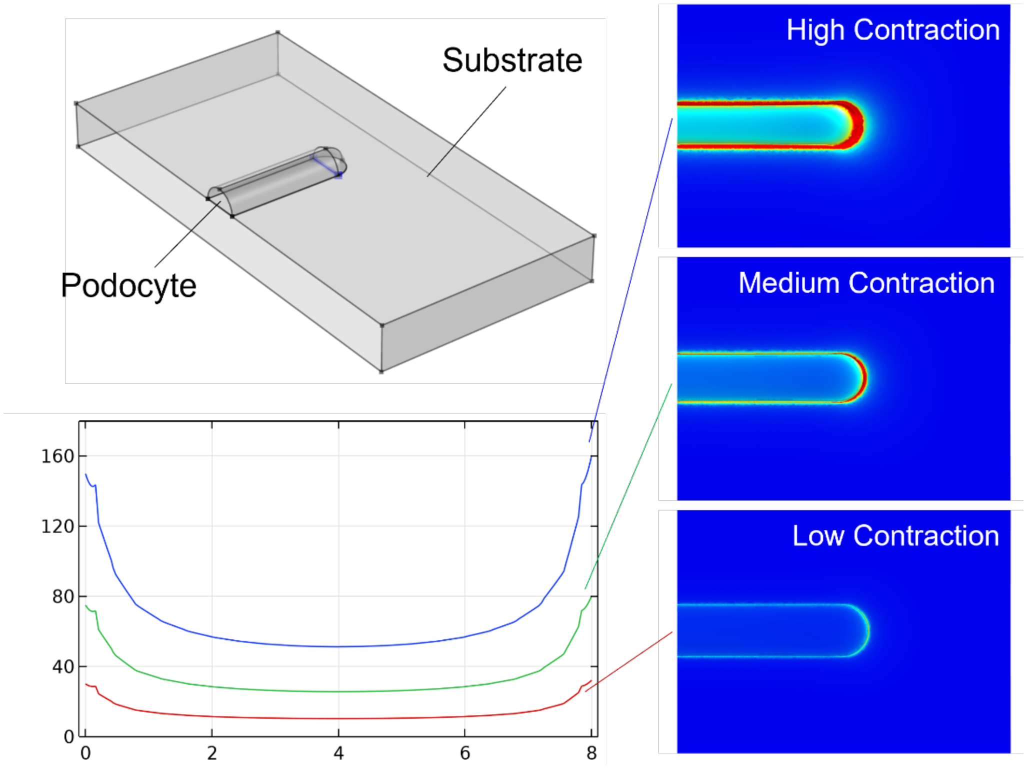

Fig. S12.
Modeling the shear stress at the adhesion surface shows dramatically reduced shear stress when the contractility inside of the cells is inhibited.

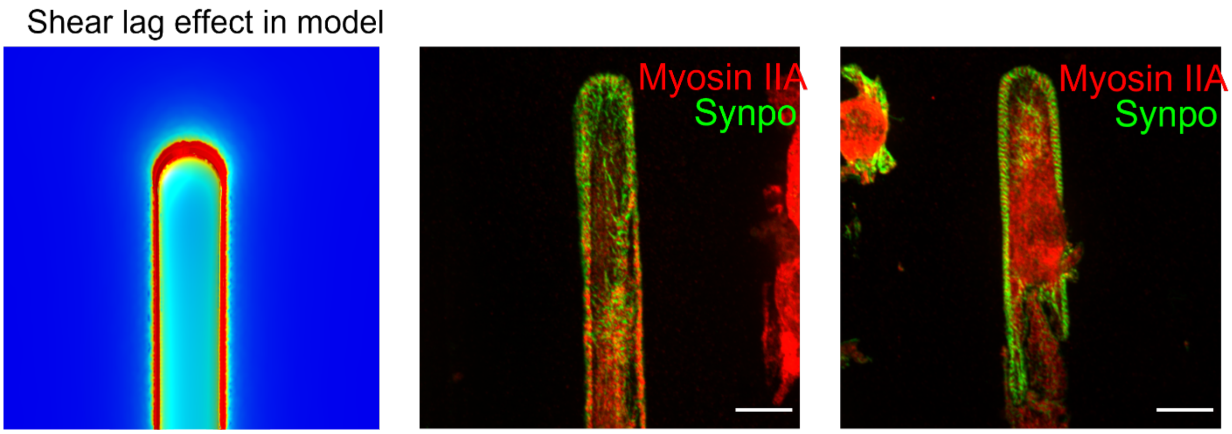

Fig. S13.

Mild myosin inhibition is not enough to eliminate all the SLSs inside the spreading podocytes, but SLSs in the center of the cells were disturbed due to shear lag effects. Scale bars, 10 µm.

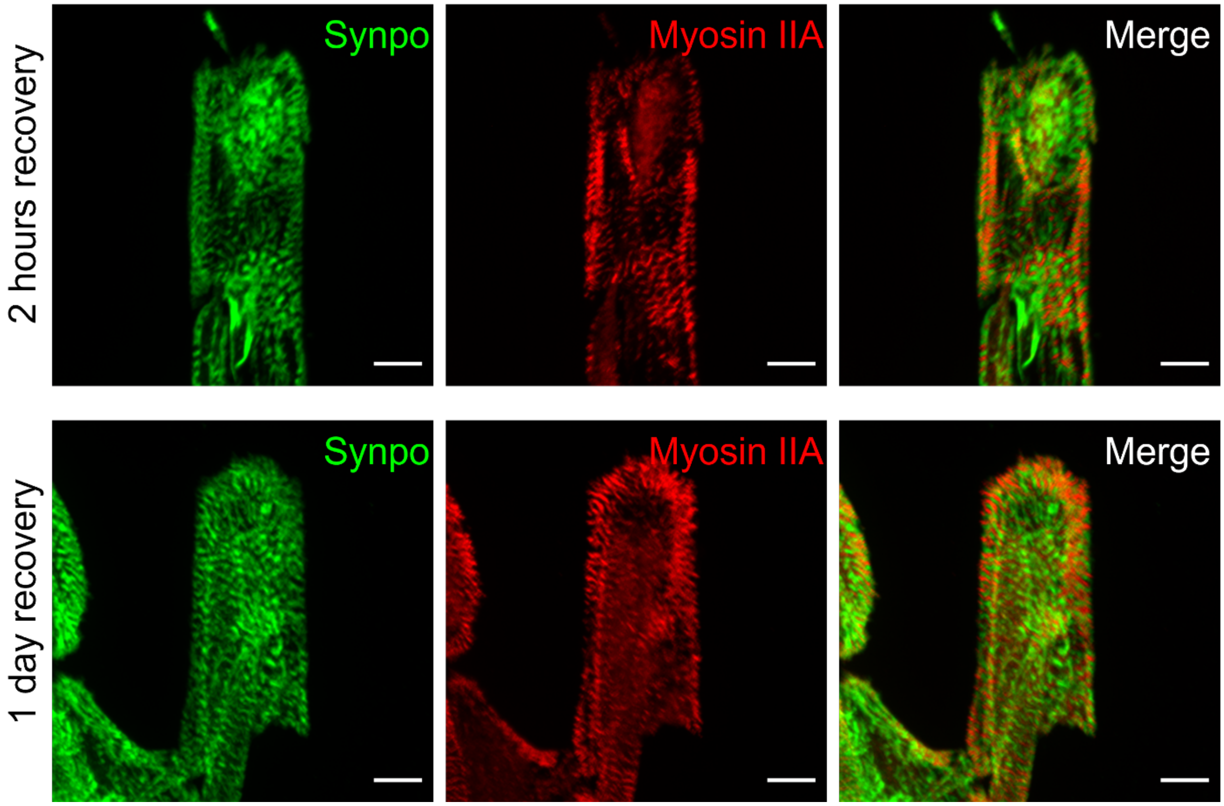

Fig. S14.

Blebbistatin wash-out led to the regeneration of SLSs in podocytes within 2 hours. Longer (1 day) recovery allowed restoration of the SLSs even at the center of the cells. Scale bars, 5 µm.
